# Proteasomal Modulation of Cellular SNAT2 (SLC38A2) Abundance and Function by Unsaturated Fatty Acid Availability*

**DOI:** 10.1074/jbc.M114.625137

**Published:** 2015-02-04

**Authors:** Francesca Nardi, Thorsten M. Hoffmann, Clare Stretton, Emma Cwiklinski, Peter M. Taylor, Harinder S. Hundal

**Affiliations:** From the Division of Cell Signalling and Immunology, Sir James Black Centre, College of Life Sciences, University of Dundee, Dundee, DD1 5EH, United Kingdom

**Keywords:** amino acid transport, fatty acid, muscle, proteasome, ubiquitin, ATF4, Amino acid transporter, Linoleic acid, SNAT2, hypertonicity

## Abstract

Expression and activity of the System A/SNAT2 (SLC38A2) amino acid transporter is up-regulated by amino acid starvation and hypertonicity by a mechanism dependent on both ATF4-mediated transcription of the *SLC38A2* gene and enhanced stabilization of SNAT2 itself, which forms part of an integrated cellular stress response to nutrient deprivation and osmotic stress. Here we demonstrate that this adaptive increase in System A function is restrained in cells subjected to prior incubation with linoleic acid (LOA, an unsaturated C18:2 fatty acid) for 24 h. While fatty acid treatment had no detectable effect upon stress-induced SNAT2 or ATF4 gene transcription, the associated increase in SNAT2 protein/membrane transport activity were strongly suppressed in L6 myotubes or HeLa cells preincubated with LOA. Cellular ubiquitination of many proteins was increased by LOA and although the fatty acid-induced loss of SNAT2 could be attenuated by proteasomal inhibition, the functional increase in System A transport activity associated with amino acid starvation/hypertonicity that depends upon processing/maturation and delivery of SNAT2 to the cell surface could not be rescued. LOA up-regulated cellular expression of Nedd4.2, an E3-ligase implicated in SNAT2 ubiquitination, but shRNA-directed Nedd4.2 gene silencing could not curb fatty acid-induced loss of SNAT2 adaptation. However, expression of SNAT2 in which seven putative lysyl-ubiquitination sites in the cytoplasmic N-terminal domain were mutated to alanine protected SNAT2 against LOA-induced proteasomal degradation. Collectively, our findings indicate that increased availability of unsaturated fatty acids can compromise the stress-induced induction/adaptation in SNAT2 expression and function by promoting its degradation *via* the ubiquitin-proteasome system.

## Teaser

**Background:** Cellular amino acid withdrawal/hypertonicity induces an adaptive increase in the expression, stability, and function of the SNAT2 amino acid transporter.

**Results:** Linoleic acid, a polyunsaturated fatty acid, suppresses the adaptive increase by targeting SNAT2 for proteolysis.

**Conclusion:** Linoleic acid promotes SNAT2 degradation *via* the ubiquitin/proteasome pathway.

**Significance:** The study provides the first evidence that SNAT2 stability is modulated by fatty acid availability.

## Introduction

SNAT1, -2, and -4 form members of the “System A” subgroup of the SLC38 family of amino acid transporters that mediate the cellular uptake of short chain neutral amino acids some of which have key roles in intermediary metabolism (*e.g.* alanine and glutamine) whereas others are nutritionally indispensable for cell function (*e.g.* methionine and threonine). Of the three System A transporters, SNAT2 (SLC38A2) is the most widely expressed but a defining feature of all members of this subgroup is their ability to mediate uptake of *N*-methylated amino acid substrates, such as 2-methylaminoisobutyric acid (Me-AIB),^2^ which has proved invaluable in their functional characterization (1). Amino acid uptake *via* all SLC38 transporters is coupled to the inward movement of sodium down its electrochemical gradient, which helps develop an outwardly-directed concentration gradient for System A substrates that can be utilized to drive the exchange uptake of a range of essential amino acids (*e.g.* leucine) through transporters (such as System L) that function in parallel with SLC38 in the plasma membrane (2, 3). This amino acid exchange arrangement is considered pivotal for sensing of amino acids upstream of mTORC1 (the mammalian target of rapamycin complex 1) and since SNAT2 itself is subject to extensive regulation by growth factors, hormones (*e.g.* IGF-1 and insulin), amino acid availability as well as osmotic stress (see reviews (1, 4)), its activity not only influences mTOR signaling (5) but controls diverse amino acid-dependent processes that impact on cell, tissue and whole body function (3).

A key conserved cellular trait is the ability of SNAT2 to be up-regulated in response to extracellular amino acid limitation. Such up-regulation is a property shared by a group of genes involved in amino acid biosynthesis and transport (*e.g.* asparagine synthase) and is normally referred to as adaptive regulation (6, 7). Sustained periods of extracellular amino acid deprivation result in up-regulation of SNAT2 expression/function by a mechanism partly sensitive to inhibitors of RNA and protein synthesis (8, 9). It should be noted that expression of other amino acid carriers and membrane transporters (*e.g.* System ASC, the Na,K-ATPase and the GLUT4 glucose transporter), are not likewise affected suggesting that the adaptive increase in SNAT2 forms part of a coordinated cell response to nutrient stress. Indeed, the transcriptional up-regulation of SNAT2 in response to amino acid withdrawal relies upon a tripartite amino acid response element in the first intron of the *SLC38A2* gene (10). Precisely how an increase in SNAT2 transcription is triggered by amino acid deficiency remains unclear, although genetic interventions and use of pharmacological inhibitors have implicated the GCN2/ATF4 pathway (7) and members of the MAP kinase family (ERK and JNK), the latter through nutrient signaling loci that remain unidentified (11, 12). While increased SNAT2 transcription contributes to the overall increase in SNAT2 abundance, we have previously shown that the SNAT2 adaptive response also includes a non-genomic component involving enhanced stabilization of the SNAT2 protein (13). It is thought that under amino acid deficient conditions SNAT2 may adopt a structurally more stable configuration, whereas SNAT2 occupancy by any one single amino acid substrate is sensed as reflecting a state of amino acid sufficiency and one that signals a reduction in SNAT2 transcription and associated destabilization/loss of SNAT2 protein (13). Consequently, SNAT2 is thought to function as an amino acid sensor or “transceptor” with the capacity to signal to nutrient responsive pathways that impact upon gene expression and protein turnover.

Although numerous studies have explored the processes by which SNAT2 is up-regulated in response to amino acid deprivation, our knowledge of the mechanisms that induce SNAT2 degradation by contrast remain poorly investigated. One potential process that may serve as an important determinant of SNAT2 turnover is the ubiquitin proteasome system (UPS) given that Nedd4.2, a ubiquitin E3-ligase, has previously been implicated in the polyubiquitination and degradation of SNAT2 (14). To our knowledge no information currently exists of how SNAT2 processing *via* the UPS may be stimulated but, given that fatty acid-derived lipids such as ceramide promote a reduction in cell surface SNAT2 with a concomitant loss in membrane transport activity (15), it is plausible that SNAT2 processing *via* the UPS may be subject to regulation by fatty acid availability. Indeed, evidence showing that fatty acids (*e.g.* oleic and linoleic acid) can stimulate proteolytic activity in muscle (16) and promote proteasomal degradation of both membrane (*e.g.* CD36 (17), tyrosinase (18)) and cytosolic proteins (*e.g.* fatty acid synthase (19)) provide strong support for this hypothesis. In this study we demonstrate that, while amino acid deprivation and hypertonicity induce a robust increase in cellular SNAT2 expression and function, the adaptive increase in SNAT2 can be significantly suppressed by pretreatment of cells with linoleic acid; a polyunsaturated (C18:2) ω-6 fatty acid. This fatty acid-induced suppression in SNAT2 adaptation cannot be attributed to reduced transcription of the *SLC38A2* gene but rather is a consequence of enhanced SNAT2 loss *via* the UPS given that the fatty acid-induced loss in SNAT2 can be attenuated by MG132, a potent cell permeable proteasomal inhibitor.

## EXPERIMENTAL PROCEDURES

### 

#### 

##### Materials

α-MEM (α-minimal essential medium), DMEM (Dulbecco's modified Eagle's medium), EBSS (Earle's Balanced Salt Solution), Opti-Mem, FBS (fetal bovine serum), and antibiotic/antimycotic solution were from Life Technologies. All other reagent-grade chemicals, including BSA and fatty acids, were obtained from Sigma-Aldrich unless otherwise stated. Fraction V fatty acid-free BSA was from Roche. Complete protein phosphatase inhibitor tablets were purchased from Boehringer-Roche Diagnostics. Anti-Slc38a2 (SNAT2) (catalogue BMP081) was from MBL, anti-ubiquitin (catalogue Z0458) was from Dako, anti-K48 (catalogue ab140601), and anti-K63 (catalogue ab179434) linkage specific ubiquitin antibodies were from Abcam, anti-α-subunit Na/K-ATPase (catalogue a6F) was from DSHB, anti-β actin(catalogue A5060), anti-GAPDH (catalogue G9545), and anti-Flag (catalogue F3165) were from Sigma, anti-Nedd4.2 and pCMV5-ubiquitin-Flag vector were obtained from Division of Signal Transduction Therapy (University of Dundee). Peptide-*N*-glycosidase F (PNGase F) was purchased from New England Biolabs. Go TaqDNA polymerase, deoxyribonucleotide phosphates (dNTPs) and Moloney Murine Leukemia Virus (M-MLV) reverse transcriptase were purchased from Promega. All primers were synthesized by the Oligonucleotide Synthesis Service (University of Dundee). X-ray film was purchased from Konica Minolta (Tokyo, Japan).

##### Design and Cloning of shRNAs

shRNA sequences were inserted into the pLKO.1-puro lentiviral vector (Sigma) as follows. Each hairpin consisted of a 21-nucleotide sense sequence, a short hairpin sequence (CTCGAG), a 21-nucleotide antisense sequence and five thymidines (a stop signal for RNA polymerase). Oligonucleotides were constructed for human Nedd4.2 (sense and antisense strands underlined, hairpin loop is indicated by italics): shRNA 1 forward *CGG*AACAATCGAACCACAACTTGG*CTCGAG*CCAAGTTGTGGTTCGATTGTT*TTTTTG*; shRNA 1 reverse *AATTCAAAAA*AACAATCGAACCACAACTTGG*CTCGAG*CCAAGTTGTGGTTCGATTGTT. Additional nucleotides were added to the ends of the oligos as shown such that annealing of the two complementary oligos resulted in overhangs consistent with those generated by EcoRI and AgeI. Oligos were annealed by mixing 2 μg of each oligo, heating to 94 °C for 10 min followed by cooling at a rate of 1 °C per min until 21 °C was reached. The final double-stranded DNA sequences were then inserted into pLKO.1-puro at the EcoRI and AgeI sites. Correct insertions of shRNA were confirmed by sequencing.

##### Lentivirus Production and Generation of Stable Nedd4.2 Knockdown HeLa Lines

Recombinant lentiviruses were produced by co-transfecting HEK 293T cells with the empty pLKO.1-puro vector, pLKO.1-puro/scramble, or pLKO.1-puro/Nedd4.2 plasmids with the envelope vector pCMV VSVg and the packaging vector pHR CMV8.2 ΔR at ratios of 3:2:2, respectively according to mass. Infectious lentiviral particles were harvested by collecting the cell culture medium 72 h later and filtered through 0.45-μm Minisart cellulose-acetate filters. Infection of HeLa cells was carried out by adding 1 ml crude lentivirus preparation to HeLa cells seeded in 6-cm dishes in the presence of 8 μg/ml polybrene. Medium was replaced with standard growth medium containing 3 μg/ml puromycin 24 h post-transduction, and cells were maintained in this selective medium until fully selected.

##### Cell Culture, Fatty Acid Treatment, and Cell Lysis

L6 skeletal muscle cells were cultured to the stage of myotubes as described previously (20) in α-minimum essential medium (α-MEM) containing 2% (*v*/*v*) FBS and 1% (*v*/*v*) antibiotic/antimycotic solution (100 units/ml penicillin, 100 mg/ml streptomycin and 250 ng/ml amphotericin B) at 37 °C with 5% CO_2_. HeLa cells were cultured in Dulbecco's Modified Eagle's Medium (DMEM), supplemented with 10% FBS, and 1% (*v*/*v*) antibiotic/antimycotic solution (100 units/ml penicillin, 100 mg/ml streptomycin, and 250 ng/ml amphotericin B), at 37 °C with 5% CO_2_. For experiments, L6/HeLa were incubated with EBSS medium supplemented with amino acid mix at 1× physiological concentration (9) and containing 2% (*w*/*v*) fatty acid-free BSA alone (control vehicle) or fatty acids that had been pre-conjugated to fatty acid-free BSA at the concentrations and for the times indicated in the figure legends, and with EBSS alone for amino acid deprivation for the penultimate 4 h of this treatment as indicated in the figure legends. Cells were rinsed before cell lysis (20) or total membranes isolation. HeLa cells were transiently co-transfected with pCDNA6-SNAT2-V5 (13) and pCMV5-ubiquitin-Flag constructs (10 μg of DNA/10-cm plate) using the polyethylenimine (PEI) method or with pCDNA6-SNAT2-V5 and pCDNA6–7A-SNAT2-V5 (a sequence in which the 7 lysine residues in SNAT2 N terminus were changed to alanine) constructs alone (3 μg DNA/6-cm plate) using lipofectamine as indicated. The 7A-SNAT2-V5 sequence was synthesized by Life Technologies Geneart and the synthesized DNA was cloned into pcDNA6-V5 plasmid *via* XhoI/XbaI digestion.

##### Isolation of Total Cell Membranes

For isolation of total membranes, cells were grown in 15 cm dishes and treated as indicated in the figure legends. Cells were washed twice with ice-cold PBS (phosphate-buffered saline) and harvested from culture dishes using a scraper. The cell suspension was centrifuged at 700 × *g* for 10 min and the resultant pellet resuspended in 3 ml of ice cold buffer I (250 mmol/liter sucrose, 20 mmol/liter HEPES, 5 mmol/liter NaN3, 2 mmol/liter EGTA, 100 μmol/liter phenylmethylsulfonyl fluoride, 10 μmol/liter trans-epoxysuccinyl-l-leucyl amido [4-guanidino] butane, 1 μmol/liter pepstatin-A, and 1 μmol/liter leupeptin; pH 7.4) and homogenized. The resulting supernatant was centrifuged at 177,000 × *g* for 1 h at 4 °C. The pellet, containing the total membranes, was resuspendend in 50 μl of Buffer I supplemented with protease inhibitor and stored at −20 °C. In some experiments, total membranes were deglycosylated using PNGase F enzyme according to manufacturer's instructions (New England Biolabs).

##### Immunoblotting

Proteins from whole cell lysates (30 μg) or total membranes (10 μg) were separated on polyacrylamide resolving gels by SDS-PAGE. Proteins were transferred onto PVDF membrane by immunoblotting as described previously (20), and blocked with 5% (*w*/*v*) milk in 0.05% (*v*/*v*) Tween 20/Tris-buffered saline for 1 h. Membranes were then incubated overnight with primary antibodies (as indicated in figure legends), and then with an appropriate peroxidase-conjugated IgG for 1 h at room temperature. Immunoreactive bands were detected by enhanced chemiluminescence on Konica Minolta X-Ray film. Quantification of immunoblots was performed using Image J software.

##### Cell Surface Biotinylation

Plasma membrane proteins were isolated using the Pierce Cell Surface Protein Isolation Kit (Thermo Scientific) as described by the manufacturer. Briefly, four 10-cm dishes of confluent HeLa cells were treated as described in the figure legend. Cells were washed with ice-cold PBS and then incubated with Sulfo-NHS-SS-Biotin solution (10 ml per dish) for 30 min at 4 °C. After the biotinylation reaction was quenched using 500 μl of quenching solution/dish, cells were scraped, pooled, and washed with 1× TBS. For lysis, cells were suspended in 500 μl of lysis buffer and sonicated. The clarified supernatant was then applied to a NeutrAvidin-agarose column, incubated for 1 h at 4 °C, and washed with wash buffer. Biotinylated membrane proteins were eluted by 400 μl of SDS-PAGE with 50 mm DTT and subject to SDS-PAGE and immunblotting with antibodies against SNAT2 and the Na,K-ATPase (a plasma membrane marker).

##### RNA Extraction and PCR

Total RNA was extracted from L6 myotubes using TRIzol® reagent according to the manufacturer's instructions (Sigma-Aldrich). Quantitative real-time PCR was carried out using a StepOnePlus Real-Time PCR System (Applied Biosystems), SYBR Green JumpStart Taq ReadyMix (Sigma-Aldrich), and primers targeting Actin and GAPDH (glyceraldehyde-3-phosphate dehydrogenase) as a control. The sequences for these primers are as follows: Slc38a2: rat forward, 5′-GCAGCCGGAGAAGGATGATGAAC-3′, reverse, 5′-GAAGAGGGCGGCAAGCAAATACA-3′, Slc38a2: human forward, 5′-GTGTTAATGGCTGTGACCCTGAC-3′, Slc38a2: human reverse, 5′- GAGACTATGACGCCACCAACTGA-3′, ATF4: rat forward, 5′-ACCCAAACCTTATGACCCACCTG-3′, ATF4: rat reverse, 5′-GCTGCTGTCTTGTTTTGCTCCATC-3′, Nedd4.2: rat forward, 5′-TCTGCCACGGACAACTACAC- 3′, Nedd4.2: rat reverse, 5′- CACAGGCCTGAGTTGGGATT-3′, Nedd4.2: human forward, 5′-CTTAGTCATCCAGTGGAGATTTGTG- 3′, Nedd4.2: human reverse, 5′-AGCAACTCCAGCTCATTTTCATC- 3′, Actin: rat forward, 5′-CCGACAGGATGCAGAAGAAG-3′, Actin: rat reverse, 5′-ATCCACATCTGCTGGAAGGTC-3′, GAPDH: forward, 5′-TGGAAAGCTGTGGCGTGAT-3′, and GAPDH: reverse 5′-GCTTCACCACCTTCTTGAT-3′. PCR amplification was performed with an initial denaturation at 95 °C for 10 min followed by 40 cycles of denaturation at 95 °C for 15 s, annealing at 55 °C for 15 s, and extension at 68 °C for 1 min. The ratio of the different genes mRNA expression to GAPDH mRNA expression was calculated as described previously (38).

##### Amino Acid Uptake

After experimental treatments, L6/HeLa were incubated with 10 μmol/liter [^14^C]Me-AIB (1 μCi/ml) for 10 min to assess the uptake of Me-AIB as previously described (20). Nonspecific tracer binding was quantified by determining cell-associated radioactivity in the presence of a saturating dose of unlabeled Me-AIB (10 mm). To terminate uptake activity, cells were washed three times with isotonic saline (0.9% NaCl, *w*/*v*) and then lysed in 50 mm NaOH. Cell-associated radioactivity was determined by liquid scintillation counting and standardized to protein concentration determined using the Bradford method (21).

##### Statistical Analyses

For multiple comparisons, statistical analysis was performed using one-way ANOVA. For individual comparisons, statistical analysis was performed using a Student's *t* test. Data analysis was performed using GraphPad Prism software and considered statistically significant at *p* < 0.05.

## RESULTS

### 

#### 

##### Linoleic Acid Represses the System A Adaptation Response

**FIGURE 1.**
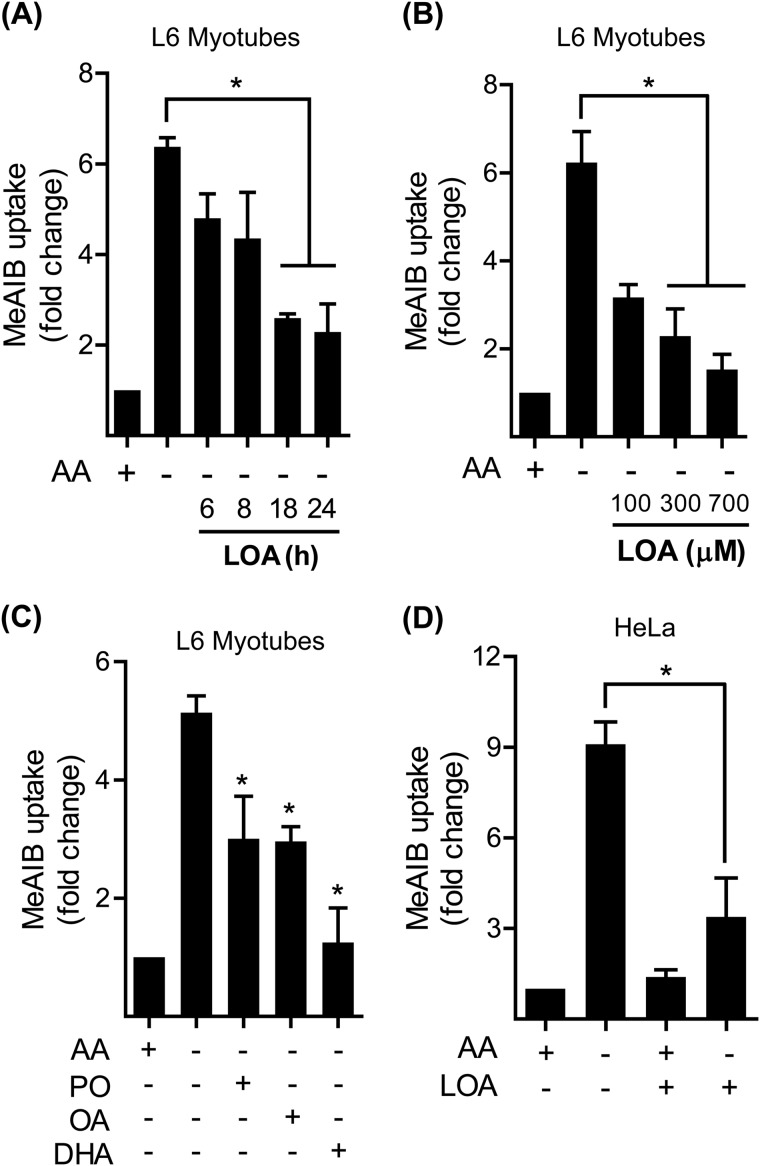
**Effects of LOA on System A transport activity in L6 myotubes and HeLa cells.** *A*, L6 myotubes were incubated with EBSS medium containing amino acids and 2% BSA + 300 μm LOA for time indicated and amino acid deprived for the last 4 h of this incubation as indicated prior to measuring MeAIB transport. *B*, L6 myotubes were incubated as in *A* + increasing concentration of LOA as indicated for 24 h and amino acid deprived for the last 4 h of this incubation prior to measuring MeAIB transport. *C*, L6 myotubes were incubated as in *A* with EBSS medium containing amino acids and 2% BSA + 300 μm palmitoleate (C16:1, PO), oleate (C18:1, OA), or docosahexaenoic acid (C22:6, DHA) for 24 h and amino acid deprived for the last 4 h of this incubation prior to measuring MeAIB transport. *D*, HeLa cells were incubated as in *A* + 300 μm linoleic acid (LOA) for 24 h and amino acid deprived for the last 4 h of this incubation prior to measuring MeAIB transport. The bar graph values are presented as mean ± S.E. of three separate experiments with *asterisks* indicating a significant change between the indicated bars (*p* < 0.05).

To assess whether System A adaptation to extracellular amino acid limitation would be sensitive to fatty acid availability, we initially monitored the effect of linoleic acid (LOA; a polyunsaturated (C18:2) ω-6 fatty acid) upon System A transport activity using Me-AIB, a paradigm non-metabolisable System A substrate. Fig. 1, *A* and *B* show that a 4 h amino acid deprivation period induces more than a 6-fold increase in System A transport in L6 myotubes compared with cells held in amino acid-containing medium. Strikingly, this adaptive increase in System A transport was reduced when myotubes were preincubated with LOA in time and dose-dependent manner: the suppressive effect of the fatty acid being maximal when myotubes were treated with LOA for 24 h at a dose of 300 μm. The ability to suppress the System A adaptive response in L6 myotubes was not confined to LOA as, in separate experiments, palmitoleic acid (C16:1), oleic acid (C18:1), or docosahexaenoic acid (C22:6) also induced a similar inhibitory response (Fig. 1*C*). Moreover, the observed suppressive effect of LOA on System A was not restricted to L6 myotubes. Fig. 1*D* shows that HeLa cells (an immortalized human adenocarcinoma cell line) also display a significant (9-fold) increase in System A adaptation upon being subjected to amino acid withdrawal, but that this is strongly suppressed when cells were pretreated with LOA.

**FIGURE 2.**
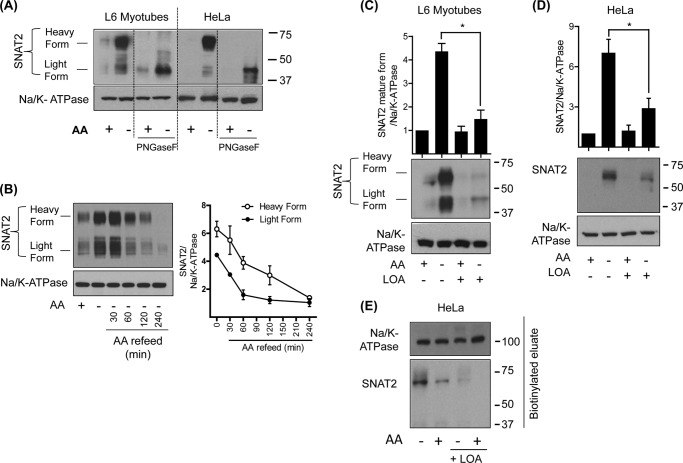
**LOA reduces SNAT2 protein abundance under amino acid deprivation in L6 myotubes and HeLa cells.** L6 myotubes and HeLa cells were incubated with EBSS medium containing amino acids and 2% BSA + 300 μm LOA for 24 h and amino acid deprived for the last 4 h of this incubation as indicated prior to total membrane isolation. In some experiments, membranes were deglycosylated using PNGaseF enzyme as indicated (*A*). Isolated total membranes from (*A*, *C*, and *D*) were subsequently immunoblotted using antibodies against SNAT2 and Na/K-ATPase. In some experiments (*B*) L6 myotubes were maintained in EBSS medium containing or lacking a physiological amino acid mix for 4 h. In some cases, myotubes deprived of amino acids were subsequently incubated with EBSS medium containing amino acids for up to 4 h. Total membranes were isolated from myotubes that had been depleted or refed amino acids and immunoblotted using antibodies against SNAT2 and the Na/K-ATPase. The blot shown in (*B*) is representative of three separate experiments. Bar graphs show quantification of blot data (mean ± S.E.) from three experiments with *asterisks* signifying a significant change (*p* < 0.05) between the indicated bars. *E*, HeLa cells were incubated with EBSS medium containing amino acids and 2% BSA + 300 μm LOA for 24 h and amino acid deprived for the last 4 h of this incubation as indicated. Using the Pierce Cell Surface Protein Isolation kit (Thermo Scientific), plasma membrane proteins were biotinylated, purified, and immunoblotted using antibodies against SNAT2 and Na/K-ATPase.

Our previous studies have indicated that the adaptive response is principally attributed to changes in cellular SNAT2 abundance (9). In line with this proposition, Fig. 2*A* shows that there is a robust increase in SNAT2 protein abundance in total membranes prepared from both L6 myotubes and HeLa cells following a 4 h amino acid deprivation period. It is noteworthy that under amino acid restricted conditions, antibodies to SNAT2 detect two distinct but broad protein bands of ∼60 kDa and ∼40 kDa, respectively, in L6 myotubes. We have previously reported that the 60 kDa band represents the mature cell surface glycosylated transporter (22), whereas the diffuse lighter band most likely reflects the partially processed but immature intracellular SNAT2 protein. Indeed, *in vitro* deglycosylation of total membranes with PNGaseF results in loss of the “heavier” immunoreactive band and detection of a more defined 40 kDa band that most likely represents the unprocessed core SNAT2 protein (Fig. 2*A*). Our findings reveal that HeLa cells also display the adaptive increase in the mature 60 kDa SNAT2 protein, but, unlike L6 myotubes, exhibit very little expression of the “lighter” form of SNAT2. However, *in vitro* deglycosylation of the mature SNAT2 from HeLa cells also generates a more defined lighter 40 kDa SNAT2 band suggesting that HeLa cells readily process new synthesized SNAT2 and retain very little of the immature SNAT2 protein within intracellular membranes such as the ER (Fig. 2*A*).

The notion that the heavy and light forms of SNAT2 represent separate membrane pools is further supported by the observation that repressing the adaptive response by cellular amino acid repletion leads to loss of each SNAT2 form at different rates. Analysis of SNAT2 content in total membranes prepared from L6 myotubes reveals that while amino acid depletion induces a 4.4-fold and 6.3-fold increase in the light and heavy forms of SNAT2, respectively, the lighter form is degraded with an half-life of ∼60 min compared with ∼120 min for the heavy form (Fig. 2*B*). The increased lag time for loss of the heavy SNAT2 form is consistent with the idea that it initially has to be internalized *via* the endosomal system before being processed for degradation, whereas as the lighter form would be directed to the proteasome immediately following synthesis and consequently degraded more rapidly.

Regardless of cell type, the adaptive increase in SNAT2 triggered by amino acid deprivation was substantially muted when either L6 myotubes or HeLa cells were pre-incubated with LOA (Fig. 2, *C* and *D*) consistent with the diminished functional System A transport capacity that is observed in cells treated with the fatty acid (Fig. 1). In line with this latter finding, it is noteworthy that a cell surface biotinylation labeling approach revealed that while amino acid depletion induces a marked increase in the heavy biotinylated SNAT2 form, this induction was severely blunted when HeLa cells were preincubated with LOA. In contrast, the abundance of biotinylated Na,K-ATPase, a well established cell surface/plasma membrane marker, was unaffected by amino acid availability or cell treatment with LOA (Fig. 2*E*).

##### Linoleic Acid Does Not Suppress the Transcriptional Up-regulation of SNAT2 Gene Expression

**FIGURE 3.**
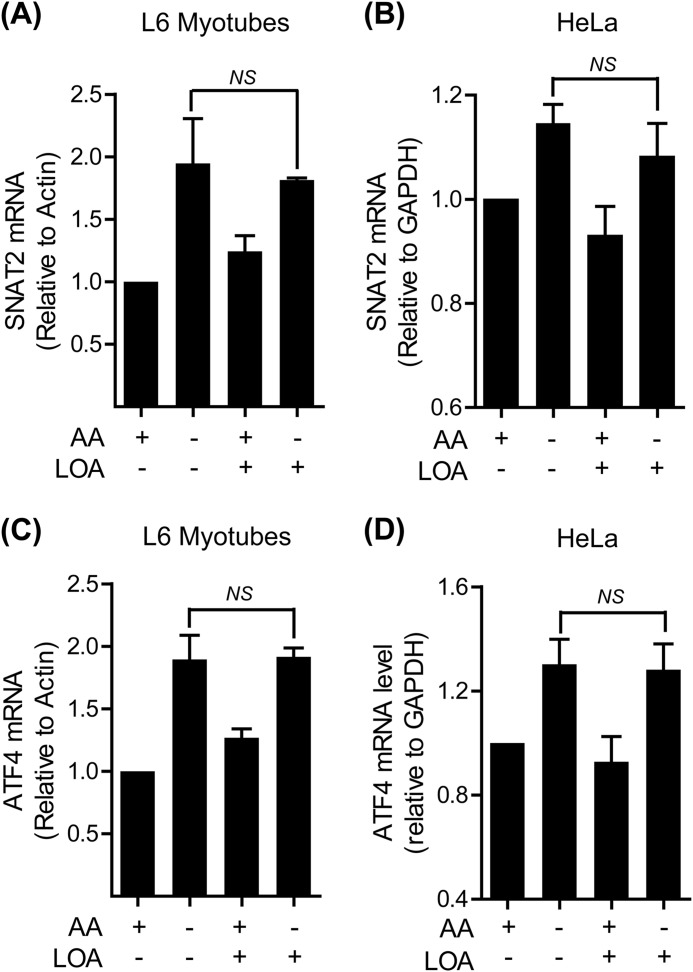
**LOA does not affect the transcriptional up-regulation of SNAT2 in L6 myotubes and HeLa cells.** *A–D*, L6 myotubes (*A*, *C*) and HeLa cells (*B*, *D*) were incubated with EBSS medium containing amino acids and 2% BSA + 300 μm LOA for 24 h and amino acid deprived for the last 4 h of this incubation as indicated. The expression of SNAT2 (*A*, *B*) and ATF4 (*C*, *D*) was tested by quantitative PCR analysis of RNA isolated from L6 myotubes (*A*, *C*) and HeLa cells (*B*, *D*). Data in the bar graphs are presented as mean ± S.E. (*n* = 3) with *NS* indicating no significant change between the indicated bars.

One possible explanation that may account for the LOA-induced reduction in SNAT2 adaptation is a failure to induce an up-regulation in *SLC38A2* gene expression. However, quantitative PCR analysis of RNA isolated from L6 myotubes and HeLa cells revealed that LOA does not significantly affect the increase in SNAT2 mRNA elicited in response to amino acid withdrawal (Fig. 3, *A* and *B*). The adaptive increase in SNAT2 gene expression is, in part, dependent upon ATF4 (7); a transcription factor whose expression, like that of the SNAT2 gene, is up-regulated in response to amino acid deficiency. Fig. 3, *C* and *D* show that ATF4 expression is increased upon cellular amino acid depletion and that it remains unaffected by pre-incubation of cells with LOA. This latter observation is fully in keeping with the increased transcription of the SNAT2 gene, an ATF4 target.

**FIGURE 4.**
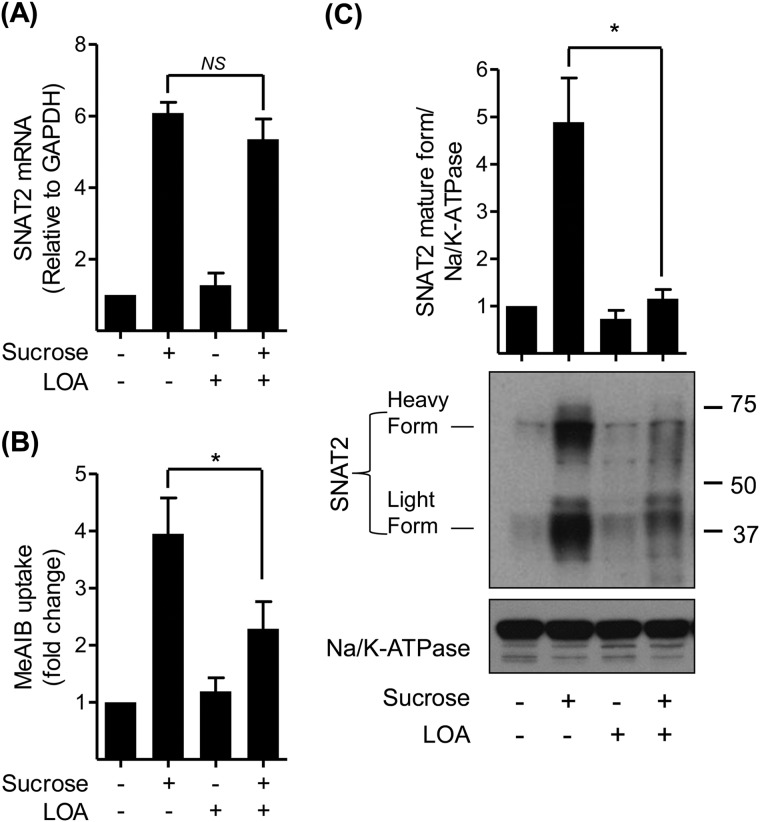
**LOA suppresses the adaptation response to hyperosmotic stress in L6 myotubes.** *A–C*, L6 myotubes were incubated with α-MEM and 2% BSA + 300 μm LOA for 24 h and with 100 mm sucrose for the last 4 h of this incubation as indicated. *A*, expression of SNAT2 was tested by quantitative PCR analysis of RNA isolated from L6 myotubes. *B*, system A/SNAT2 activity was assayed by measuring the uptake of MeAIB. *C*, total membranes were isolated and immunoblotted using antibodies against SNAT2 and Na/K-ATPase. Data in the bar graphs are presented as mean ± S.E. (*n* = 3) with *asterisks* indicating a significant change (*p* < 0.05) between the indicated bars.

System A expression/activity is also known to be transcriptionally up-regulated in response to cellular hyperosmotic stress, although the signaling mechanisms that drive this increase in SNAT2 gene expression are considered distinct from those that are responsive to amino acid withdrawal (12). Given the ability of LOA to suppress the functional increase in SNAT2/System A activity in cells subjected to amino acid starvation we assessed if the fatty acid could also perturb the up-regulation of SNAT2 expression/function normally induced by hyperosmotic stress. Fig. 4*A* shows that L6 myotubes exposed to sucrose-induced hypertonic stress exhibit a 6-fold increase in SNAT2 mRNA expression and that this induction was unaffected in myotubes pretreated with LOA. However, subsequent analysis of System A function reveals that while System A-mediated Me-AIB uptake was elevated by ∼4-fold in osmotically shocked cells, it was significantly blunted in LOA-treated myotubes (Fig. 4*B*). The observed loss in transport capacity tallies with loss of the mature (cell surface) and immature SNAT2 protein as judged by immunoblotting total membranes prepared from L6 myotubes with SNAT2 antibodies (Fig. 4*C*).

##### Regulation of SNAT2 Protein Abundance

**FIGURE 5.**
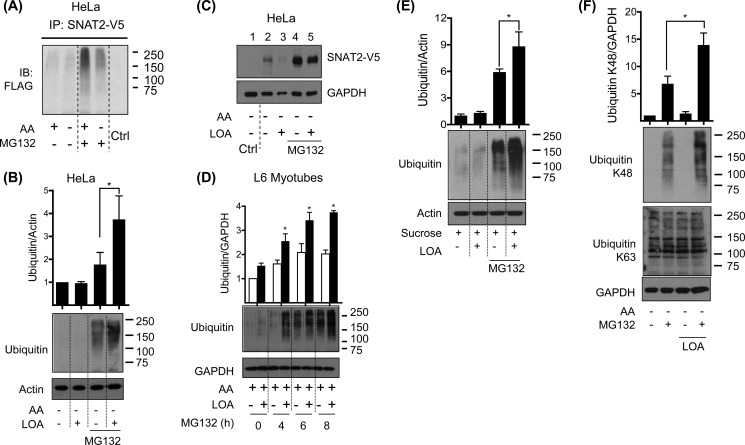
**LOA induces the ubiquitin proteasomal system under amino acid withdrawal and hyperosmotic shock in L6 myotubes and HeLa cells.** *A*, HeLa cells were transiently co-transfected with SNAT2-V5 and ubiquitin-Flag plasmids by PEI method. 48 h post-transfection cells were incubated with EBSS medium containing amino acids for 8 h and amino acid deprived for the last 4 h of this incubation in medium containing or lacking MG132 (10 μm). SNAT2 was immunoprecipitated from whole cell lysates using an anti-V5 antibody and then immunoblotted using antibody against Flag. *B*, HeLa cells were incubated with EBSS medium containing amino acids and 2% BSA + 300 μm LOA for 24 h and amino acid deprived for the last 4 h of this incubation in medium containing or lacking MG132 (10 μm) as indicated. *C*, HeLa cells were transiently transfected with SNAT2-V5 by using Lipofectamine. 24 h post-transfection cells were incubated with EBSS medium containing amino acids and 2% BSA + 300 μm LOA for 24 h and amino acid deprived for the last 4 h of this incubation in medium containing or lacking MG132 (10 μm). *D*, L6 myotubes were incubated with EBSS medium containing amino acids and 2% BSA + 300 μm LOA for 24 h and treated with MG132 (10 μm) for the penultimate hours of this incubation as indicated. *E* and *F*, L6 myotubes were incubated with α-MEM (*E*) or EBSS medium containing amino acids (*F*) and 2% BSA + 300 μm LOA for 24 h and treated with 100 mm sucrose (*E*) or amino acid deprived (*F*) for the last 4 h of this incubation in medium containing or lacking MG132 (10 μm). Cell lysates from *B–F* were immunoblotted using antibodies against the proteins indicated. Immunoblotted are representative of three (*A*) and two (*C*) distinct experiments. Data in the bar graphs are presented as mean ± S.E. (*n* = 3) with *asterisks* indicating a significant change (*p* < 0.05) between the indicated bars (*B*, *E*, *F*) or between the filled and unfilled bars (*D*).

The data presented in Figs. 2–4 support the idea that SNAT2 protein abundance may be specifically modulated in response to LOA availability. Since SNAT2 stability has previously been shown to be regulated by the ubiquitin proteasome system (UPS) (14), we subsequently explored whether LOA may promote SNAT2 degradation via this proteolytic pathway. Consistent with the idea that SNAT2 ubiquitination/degradation would be reduced in amino acid deprived cells (*i.e.* those exhibiting an adaptive increase in SNAT2), we find that HeLa cells transiently cotransfected with plasmids containing a CMV-driven SNAT2 construct (containing a C-terminal V5-His_6_ epitope tag) and Flag-ubiquitin exhibit reduced SNAT2 ubiquitination compared with cells maintained in amino acid containing medium under conditions when the UPS is inhibited using MG132 (Fig. 5*A*). To assess the impact of LOA on cellular ubiquitination under amino acid-deprived conditions, whole cell lysates from HeLa cells incubated with the fatty acid for 24 h were immunoblotted with anti-ubiquitin antibodies. Fig. 5*B* shows that the presence of MG132 is required for detection of accumulated ubiquitinated proteins and that HeLa cells incubated with LOA exhibit a significant increase in protein ubiquitination when the signal intensity is quantified along the vertical length of the gel lane using Image J software (Fig. 5*B*, *upper panel*). Intriguingly, while we can detect expression of transiently expressed SNAT2-V5 in HeLa cells under amino acid deprived conditions the abundance of the V5-tagged transporter is considerably elevated upon inhibiting the UPS with MG132 (Fig. 5*C*, compare *lanes 2* and *4*). Importantly (and in line with data already presented) LOA induces a marked loss in SNAT2-V5 protein (compare *lanes 2* and *3*) that was not observed when MG132 was present (compare *lanes 4* and *5*).

The LOA-induced increase in protein ubiquitination is not restricted to HeLa cells. Fig. 5*D* shows that, in the presence of MG132, L6 myotubes also exhibit the classic ubiquitin smear widely accepted as a typical biochemical signature for protein ubiquitination. The intensity of this ubiquitin smear was significantly enhanced in myotubes treated with LOA and, as expected, was further elevated with prolonged exposure to MG132 as this would contribute to increased accumulation of ubiquitinated proteins (Fig. 5*D*). Since LOA also attenuates increases in SNAT2 protein that occurs in response to hypertonic stress, it was not all together surprising to discover that the fatty acid enhances protein ubiquitination in myotubes subjected to a 4 h hyperosmotic challenge with sucrose (Fig. 5*E*). To further substantiate the idea that LOA can induce proteasomal degradation of cellular proteins, such as SNAT2, we monitored the effect of LOA on lysine 48 (K48) or 63 (K63)-specific ubiquitination using linkage-specific antibodies. K48-ubiquitin chains primarily target proteins for proteasomal degradation (24), whereas formation of K63-ubiquitin chains direct proteins to alternative fates such as trafficking to the lysosome, participation in intracellular signaling and DNA repair in a manner unaffected by proteasomal inhibitors (25). Fig. 5*F* shows that, while LOA induces a significant gain in cellular K48-protein ubiquitination, we were unable to detect any notable changes in the K63-protein ubiquitination pattern irrespective of whether cells had been incubated with MG132 or not.

##### Effects of LOA and Proteasomal Inhibition on SNAT2 Protein and System A Transport

**FIGURE 6.**
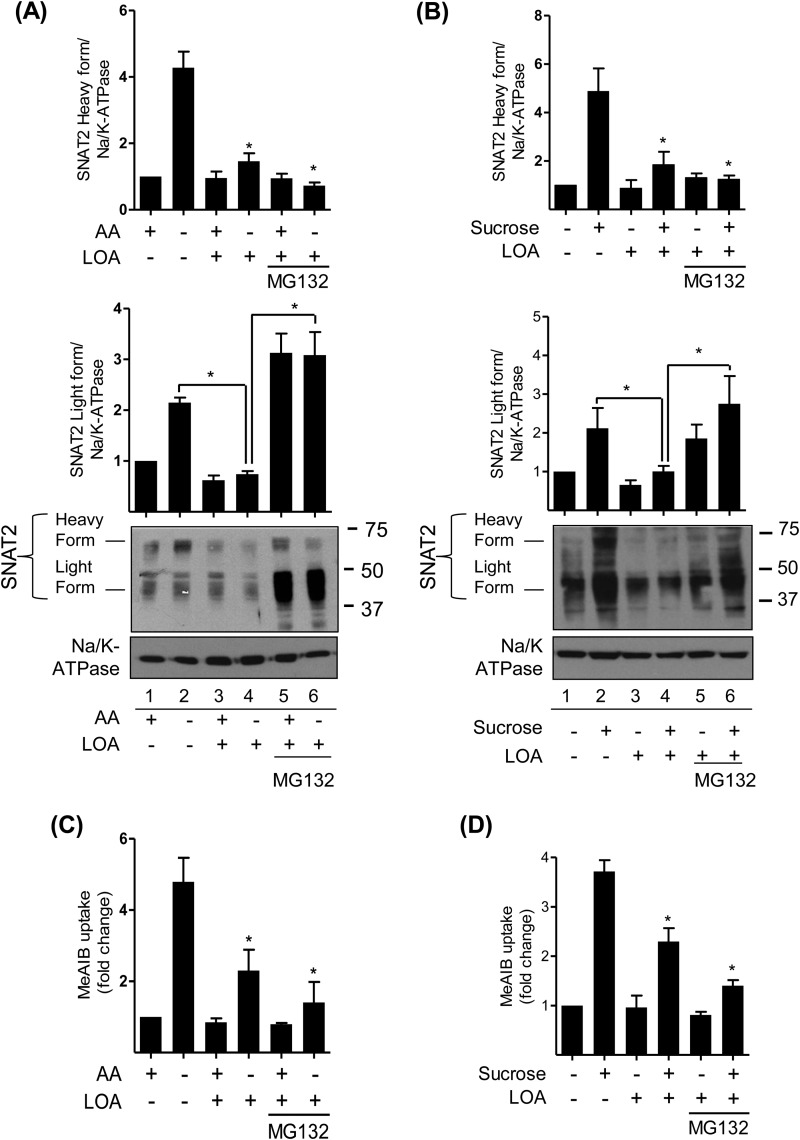
**LOA targets SNAT2 immature form for proteasomal degradation under amino acid withdrawal and hyperosmotic shock in L6 myotubes.** *A–D*, L6 myotubes were incubated with EBSS medium containing amino acids (*A*, *C*) or in α-MEM (*B*, *D*) and 2% BSA + 300 μm LOA for 24 h. During the last 4 h of this incubation cells were either retained in EBSS buffer containing or lacking amino acid (*A*, *C*) or held in α-MEM medium supplemented or not with 100 mm sucrose (*B*, *D*). *A* and *B*, total membranes were isolated and subsequently immunoblotted using antibodies against SNAT2 and Na/K-ATPase. *C* and *D*, system A/SNAT2 activity was assayed by measuring the uptake of MeAIB. Data in the bar graphs are presented as mean ± S.E. (*n* = 3) with *asterisks* indicating a significant change (*p* < 0.05) compared with the amino acid deprived (*C*) or the sucrose stimulated (*D*) alone.

Having established that LOA can promote a net increase in cellular protein ubiquitination (Fig. 5) and that MG132 can halt LOA-induced loss of SNAT2 protein (Fig. 5*C*), we subsequently monitored the effect of LOA and MG132 on SNAT2 stability and function in L6 myotubes subjected to amino acid deprivation and hypertonic stress. Fig. 6, *A* and *B* show that a 4-h period of extracellular amino acid depletion or hypertonic stress induces a 4–5-fold increase in the expression of the mature cell surface SNAT2 protein, which is associated with a significant (3–5-fold) increase in Me-AIB uptake under both circumstances (Fig. 6, *C* and *D*). However, pretreatment of L6 myotubes with LOA for 24 h prior to amino acid depletion or hypertonic stress during the penultimate 4 h LOA incubation period significantly restrains the increase in the abundance of both the light and heavy SNAT2 forms (Fig. 6, *A* and *B*) as well as the associated stimulus-induced increase in System A transport activity (Fig. 6, *C* and *D*). Intriguingly, cell treatment with MG132 not only halted the LOA-induced loss of the lighter SNAT2 form but induced its accumulation, whereas the inhibitor was unable to protect against loss of the mature (cell surface) SNAT2 protein. The finding that Me-AIB uptake was not enhanced in cells that were amino acid depleted or those subject to hypertonic stress when having been pre-incubated with LOA and MG132 is fully consistent with loss of the cell surface SNAT2 protein.

##### Modulation of Nedd4.2 Expression by LOA

**FIGURE 7.**
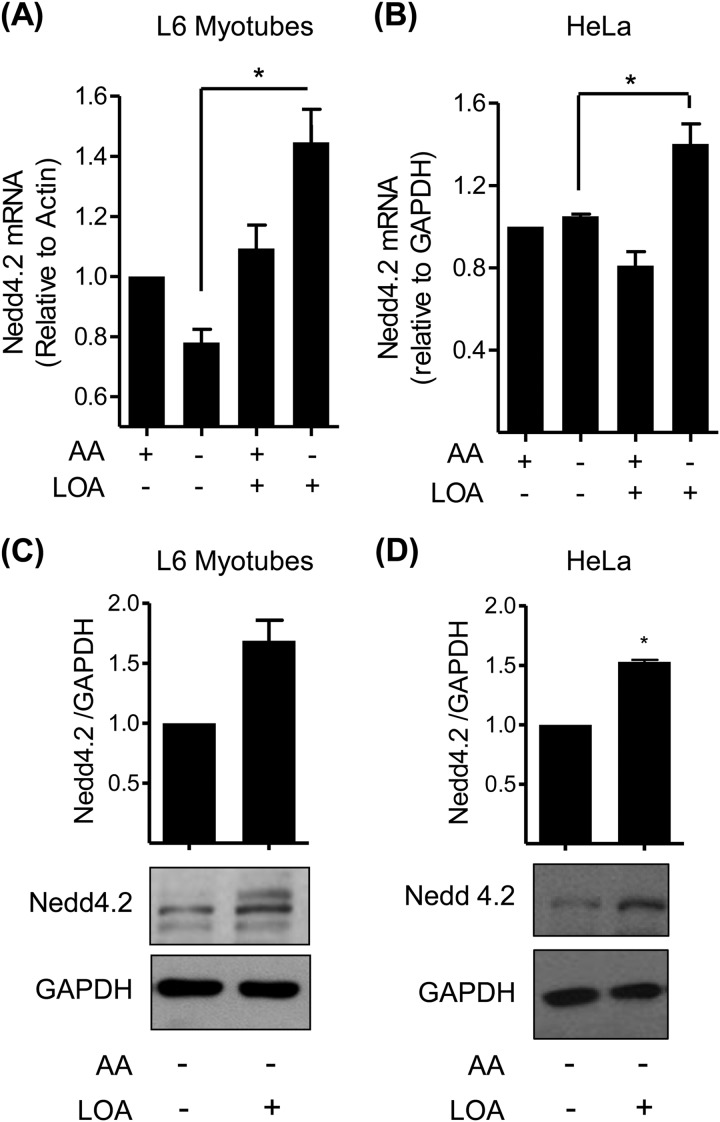
**LOA enhances Nedd4.2 mRNA and protein level in L6 myotubes and HeLa cells.** *A–D*, L6 myotubes (*A*, *C*) and HeLa cells (*B*, *D*) were incubated with EBSS medium containing amino acids and 2% BSA + 300 μm LOA for 24 h and amino acid deprived for the last 4 h of this incubation as indicated. *A* and *B*, expression of Nedd4.2 was tested by quantitative PCR analysis of RNA isolated from L6 myotubes (*A*) and HeLa cells (*B*). *C* and *D*, cell lysates were immunoblotted using antibodies against Nedd4.2 and GAPDH. Data in the bar graphs are presented as mean ± S.E. (*n* = 3) with *asterisks* indicating a significant change (*p* < 0.05) between the indicated bars (*A*, *B*) or compared with the control (*C*, *D*).

**FIGURE 8.**
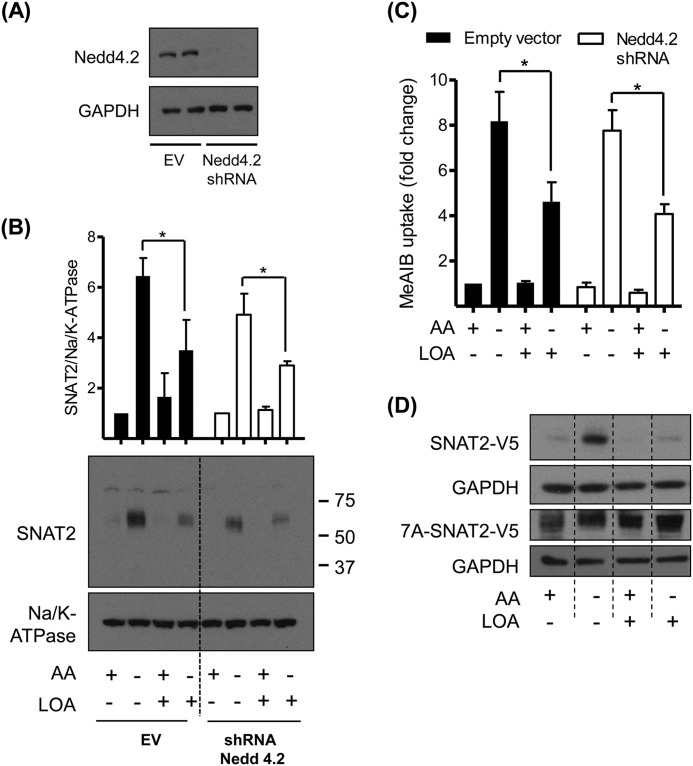
**LOA suppressive effect upon SNAT2 adaptation response is not mediated by Nedd4.2 in HeLa cells.** *A–C*, HeLa cells stably expressing empty vector or shRNA targeting Nedd4.2 were incubated with EBSS medium containing amino acids and 2% BSA + 300 μm LOA for 24 h and amino acid deprived for the last 4 h of this incubation as indicated. *A* and *B*, cell lysates (*A*) and isolated total membranes (*B*) were immunoblotted using antibodies against the proteins indicated. *C*, system A/SNAT2 activity was assayed by measuring the uptake of MeAIB. *D*, HeLa cells were transiently transfected with SNAT2-V5 or 7A-SNAT2-V5 mutant by using lipofectamine. 24 h post-transfection cells were incubated with EBSS medium containing amino acids and 2% BSA + 300 μm LOA for 24 h and amino acid deprived for the last 4 h of this incubation. Cell lysates were immunoblotted using antibodies against V5 and GAPDH. Immunoblots are representative of three (*A–C*) and two (*D*) distinct experiments. Data in the bar graphs are presented as mean ± S.E. (*n* = 3), with *asterisks* indicating a significant change (*p* < 0.05) between the indicated bars (*B*, *C*).

One potential modifier of SNAT2 stability is Nedd4.2, an E3-ubiquitin ligase that has previously been implicated in the turnover of SNAT2 *via* the UPS (14). Analysis of Nedd4.2 mRNA expression in both L6 myotubes and HeLa cells revealed that while amino acid availability *per se* had no effect on Nedd4.2 gene expression, cells treated with LOA under amino acid deprived conditions exhibit a significant increase in both Nedd4.2 mRNA and protein abundance (Fig. 7, *A–D*). To further investigate if this LOA-induced increase in Nedd4.2 contributes to the destabilization/functional loss of SNAT2, we generated HeLa cells in which expression of Nedd4.2 was stably silenced using shRNA. Fig. 8*A* shows that, compared with control cells transfected with the empty lentiviral vector, Nedd4.2 protein was undetectable in cells expressing shRNA targeting Nedd4.2 mRNA. However, despite the effective silencing of Nedd4.2 expression we observed little impact upon the adaptive increase in SNAT2 that occurs following amino acid withdrawal or upon the ability of LOA to curb the increase in both SNAT2 protein (Fig. 8*B*) and System A transport activity (Fig. 8*C*).

We have previously indicated that the cytoplasmic N-terminal domain of SNAT2 plays an important role with respect to stability of the transporter (13). While the ubiquitination sites on SNAT2 have not yet been mapped, of the 12 cytosolic exposed lysine residues that may potentially be ubiquitinated, seven are located within the N-terminal tail. Thus, one or more of these lysine residues may influence SNAT2 stability. To test this possibility we mutated all 7 lysine residues in the N-terminal tail to alanine (7A mutant) and expressed the mutated 7A-SNAT2-V5 construct from a CMV-non amino acid responsive promoter. Fig. 8*D* shows that while HeLa cells expressing wild-type SNAT2-V5 exhibit stabilization of SNAT2 protein in amino acid deprived cells, and that this is not apparent in cells treated with LOA, the 7A-SNAT2-V5 appears to be equally stable under all conditions tested.

## DISCUSSION

The work presented herein demonstrates that sustained cell incubation with LOA perturbs the adaptive up-regulation of System A/SNAT2 that is normally instituted when cells are subjected to amino acid deprivation or a hypertonic-inducing stress stimulus. The up-regulation of SNAT2 in response to such stimuli is not just a consequence of increased transcription of the *SLC38A2* gene (10) but also involves greater stabilization of the SNAT2 protein itself (13). However, our observations indicate that while the increase in SNAT2 mRNA expression triggered by extracellular amino acid deficiency or hypertonicity is unaffected, the resultant increase in SNAT2 protein and transport activity fail to materialize in cells preincubated with LOA because the transporter is targeted for degradation by the UPS. The ability of fatty acids to modulate SNAT2 abundance *via* this proteolytic route has not, to our knowledge, been previously documented and provides an additional facet to the biology of one of the most extensively regulated mammalian amino acid transporters known. However, it is important to stress that, while the adaptive increase in SNAT2 abundance is diminished by LOA, the fatty acid also increases the total content of ubiquitinated proteins in cells (Fig. 5, *D* and *E*) suggesting that SNAT2 is likely to be one of many proteins targeted to the UPS in response to increased fatty acid availability. The physiological significance of such regulation of the UPS by fatty acids remains unclear but may form part of a mechanism diverting use of key amino acids for alternative metabolic fates in times of need. The effects of LOA on SNAT2 function/turnover in our muscle cells was apparent over the 0.1–0.3 mm range. Recent work has shown that the concentration of plasma free fatty acids increases from ∼0.2 mm to ∼0.8 mm in human subjects after 10 h of fasting as a result of increased lipolysis (26). This increased lipolytic activity not only furnishes tissues, such as skeletal muscle, with fatty acids for use as metabolic fuel during fasting, but, *via* activation of UPS-mediated proteolysis, will liberate amino acids, such as alanine, into the circulation for use in hepatic gluconeogenesis and energy metabolism. Moreover, it is also conceivable that the fatty acid-induced loss in cell surface SNAT2 that we see in muscle cells ensures that once released, alanine, a SNAT2 substrate, is not taken back up by muscle from the circulation.

Precisely how unsaturated fatty acids stimulate the UPS remains unclear, although in rat skeletal muscle they are known to stimulate the proteolytic activity of 20 S proteasomes (16). Accelerated skeletal muscle protein degradation is also a feature in mice fed a high fat diet, where enhanced *in vivo* muscle proteolysis has been linked to increased expression of two E3 ubiquitin ligases (atrogin-1/muscle atrophy F-box (MAFbx) and muscle RING finger-1 (MuRF1)) (27), that are closely associated with muscle protein degradation (28). Although there is no evidence in the literature linking MAFbx or MuRF1 to regulation of SNAT2 turnover, we postulated that Nedd4.2 may be a prime candidate in the fatty acid induced degradation of SNAT2 given that this E3 ligase has been implicated in the ubiquitination of the transporter in 3T3-L1 adipocytes (14). However, our data indicate that while LOA induces up-regulation of Nedd4.2 in amino acid-deprived cells in a manner that would be consistent with the observed reduction in SNAT2 triggered by LOA, cellular depletion of Nedd4.2 (using shRNA) is unable to prevent the loss in SNAT2 protein caused by the fatty acid. This latter observation, along with the finding that we do not observe any net increase in SNAT2 abundance in cells lacking Nedd4.2 *vis a vis* control cells (*i.e.* those infected with the empty lentiviral vector), would imply that Nedd4.2 is dispensable with respect to regulation of SNAT2 turnover *via* the UPS, at least in the cells used in our study.

**FIGURE 9.**
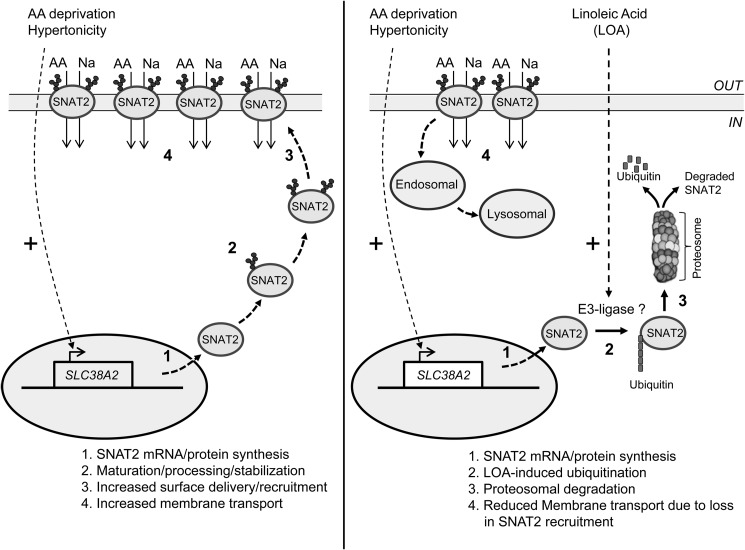
**Scheme illustrating the cellular regulation of SNAT2 in response to amino acid withdrawal or hypertonicity in the absence and presence of cell pretreatment with linoleic acid.**

Ectopic expression of EGFP-labeled SNAT2 (*aka* ATA2) in 3T3-L1 adipocytes has been used as a strategy for investigating SNAT2 ubiquitination, and turnover and it is noteworthy that proteasomal inhibition within these cells using MG132-induced marked perinuclear accumulation of the transporter (14). Intriguingly, in these cells MG132 also increased adipocyte Me-AIB uptake which, coupled with the observation that Nedd4.2 partially colocalized with SNAT2 at the cell surface, led the authors of the study to conclude that surface SNAT2 would normally be internalized and degraded by the UPS (14). While there is some evidence that such a mechanism may account for the regulation and turnover of certain plasma membrane proteins (17, 29, 30), most are internalized and undergo trafficking to early endosomes and sorting to multivesicular bodies prior to lysosomal fusion and proteolysis (31, 32). Our studies assessing the effect of LOA on the SNAT2 adaptive response lead us to propose that SNAT2 may have two potential proteolytic fates depending on its cellular localization. It is our view that LOA targets the immature intracellular SNAT2 protein for degradation by the UPS, whereas the mature surface form is likely to be degraded by a non-UPS route (Fig. 9). This proposition is based on our finding that MG132 not only curtails loss of immature SNAT2 ear-marked for degradation by LOA, but promotes its intracellular accumulation, much like that seen in adipocytes expressing EGFP-labeled SNAT2 (14). However, since MG132 will have no direct inhibitory effect on LOA-induced protein ubiquitination, we contest that the normal processing, maturation, and delivery of SNAT2 to the cell surface (which represents an integral part of the System A adaptive response) is likely to be arrested by its ubiquitination. Consistent with this view, MG132 fails to promote any buildup of the mature (cell surface) SNAT2 form in muscle cells and as a consequence System A transport activity cannot be raised to the level induced by amino acid deprivation or hypertonic stress as occurs in cells not treated with LOA and MG132 (Fig. 6, *C* and *D*). In addition, it is noteworthy that, while Me-AIB uptake in cells maintained in an amino acid sufficient environment is normally low, LOA *per se* has no impact on basal amino acid uptake or upon the abundance of the mature/heavy SNAT2 protein although the fatty acid is still capable of causing a modest reduction in the abundance of the immature SNAT2 protein (compare *lanes 1* and *3* in Fig. 6, *A* and *B*).

We have previously indicated that the cytoplasmic domains of SNAT2 are the most likely candidate regions for associating with the proteolytic machinery and therefore most likely to confer amino acid-regulated stability effects (13). Consistent with this idea, we have already shown that grafting the N-terminal tail of SNAT2 onto SNAT5 (a related family member that does not exhibit adaptation to amino acid withdrawal) renders the stability of the SNAT5 protein sensitive to amino acid availability (13). Closer inspection of the cytoplasmic SNAT2 N-terminal tail reveals the presence of 7 lysine residues that may potentially serve as ubiquitin conjugation sites. The finding that transfection of HeLa cells with a SNAT2-V5 construct in which all 7 lysine residues were mutated to alanine results in expression of a SNAT2 protein that is constitutively more stable than wild-type SNAT2 and one that is protected against LOA-induced proteolysis would tend to support our proposition. Key unresolved issues include the identity of the E3-ligase that promotes ubiquitination of one or more of these lysine residues and the mechanism by which LOA enhances the SNAT2-ubiquitin tagging process. While increased expression of E3 ligases in response to unsaturated fatty acids may serve to drive ubiquitination/degradation of cellular proteins, the signaling events responsible for sensing and initiating this response remain poorly understood. However, two potential pathways that may contribute to fatty acid induced activation of the UPS include signaling initiated by TLR4, a member of the Toll-like family of receptors, and peroxisome proliferator-activated receptor α (PPARα), which functions as a major regulator of lipid metabolism in tissues such as liver and muscle (33). TLR4 activation can be typically initiated by binding of lipopolysaccharide (LPS) but also by fatty acids (34, 35) and, as such, activation of the receptor by LPS has been linked to up-regulation of both MAFbx and MuRF1 and increased muscle atrophy by the UPS (36). Whether TLR4 signaling may account for LOA-induced SNAT2 ubiquitination is currently unknown, but in separate experiments we find that treatment of L6 myotubes with LPS induces TLR4 signaling without any significant changes in System A mediated Me-AIB uptake (data not shown). Although this latter finding tends to negate involvement of the TLR4 signaling axis as a specific regulator of SNAT2 turnover, it does not discount the possibility that it may stimulate loss of other cellular proteins by the UPS. Much like TLR4, PPARα also binds a variety of fatty acid ligands including LOA and recent work has highlighted that activation of PPARα both increases content of ubiquitinated proteins in rat skeletal muscle and stimulates proteolytic flux through the UPS with a consequential increase in muscle atrophy (37). As with TLR4 activation, the increase in protein ubiquitination associated with ligand-induced activation of PPARα involves higher muscle expression of MAFbx and MuRF1. While the impact of PPARα activation upon SNAT2 turnover has not been directly investigated, it is noteworthy that increases in SNAT2 abundance in response to chronic insulin stimulation can be fully repressed by PPARα activation in human trophoblasts (23). Whether this PPARα-linked repression is due to enhanced proteasomal degradation of SNAT2 or the result of impaired insulin signaling events that participate in the up-regulation of SNAT2 expression is currently unknown, but testing these possibilities represent important investigative goals of future work.

In summary, the present work has demonstrated that the adaptive up-regulation of SNAT2 that is normally instigated in response to cellular amino acid depletion or hypertonic stress can be attenuated by prior exposure of cells to unsaturated fatty acids, such as LOA. This fatty acid-induced reduction is not due to suppression of SNAT2 gene transcription that is initiated by the amino acid withdrawal or hypertonic stimulus, but to enhanced destabilization and degradation of intracellular SNAT2 by the UPS. As a consequence, delivery of newly synthesized/processed SNAT2 to the cell surface is substantially diminished resulting in a concomitant loss in System A transport activity, which will have implications for cellular adaptation to reduced extracellular amino acid availability and hypertonicity.

## Footnotes

2 The abbreviations used are:

Me-AIB: 2-methylaminoisobutyric acid
LOA: linoleic acid
mTORC: mammalian target of rapamycin complex
UPS: ubiquitin proteasome system.
